# Molecular biochemical aspects of salt (sodium chloride) in inflammation and immune response with reference to hypertension and type 2 diabetes mellitus

**DOI:** 10.1186/s12944-021-01507-8

**Published:** 2021-08-01

**Authors:** Undurti N. Das

**Affiliations:** UND Life Sciences, 2221 NW 5th St, Battle Ground, WA 98604 USA

**Keywords:** Hypertension, diabetes mellitus, inflammation, T cells, reactive oxygen species, sodium, potassium

## Abstract

Obesity, insulin resistance, type 2 diabetes mellitus (T2DM) and hypertension (HTN) are common that are associated with low-grade systemic inflammation. Diet, genetic factors, inflammation, and immunocytes and their cytokines play a role in their pathobiology. But the exact role of sodium, potassium, magnesium and other minerals, trace elements and vitamins in the pathogenesis of HTN and T2DM is not known. Recent studies showed that sodium and potassium can modulate oxidative stress, inflammation, alter the autonomic nervous system and induce dysfunction of the innate and adaptive immune responses in addition to their action on renin-angiotensin-aldosterone system. These actions of sodium, potassium and magnesium and other minerals, trace elements and vitamins are likely to be secondary to their action on pro-inflammatory cytokines IL-6, TNF-α and IL-17 and metabolism of essential fatty acids that may account for their involvement in the pathobiology of insulin resistance, T2DM, HTN and autoimmune diseases.

## Introduction

Both type 2 DM (T2DM) and hypertension (HTN) are common diseases in almost all countries of the world. It is estimated that about 50% of the population above the age of 45 years have HTN and another 10-20% of the population have T2DM (detected or undetected). Almost 50% of the patients who have T2DM have or develop HTN eventually. Thus, both T2DM and HTN are present in many of the patients (population). In addition, obesity is also common. Patients with both T2DM and HTN are generally obese. It is noteworthy that obesity, T2DM and HTN are all associated with insulin resistance. In fact, insulin resistance is the first to develop in those who eventually develop HTN and T2DM. It is now recognised that obesity, T2DM and HTN are all low-grade systemic inflammatory conditions. Coronary heart disease (CHD) eventually occurs in some, if not all, of the patients with insulin resistance, obesity, T2DM and HTN. Thus, all these conditions have overlapping features and one condition may lead to the other(s) [1–8]. Insulin resistance seems to be a common factor underlying all these conditions.

Of all the dietary factors that are known to influence the development of HTN and T2DM, salt, potassium, essential fatty acids, minerals, trace elements, vitamins and calorie intake are important. Salt does not influence the development of HTN in all but in a subset of patients who have salt sensitive HTN, whereas potassium seems to suppress the pro-hypertensive action of salt [9, 10]. In addition to these dietary factors, exercise is also an equally important factor in the prevention and management of hypertension and T2DM. Sodium (salt) is an essential nutrient in man and physiological need in adults is only of the order of 8–10 mmol/day (184–230 mg/day). It may be noted that the equivalence between sodium and salt is as follows: 1g sodium chloride = 17.1 mmol or 393.4 mg sodium. Salt intake induces a significant difference in the prevalence of hypertension. Higher intake of salt is expected to produce substantial increase in blood pressure, though some studies did not reproduce this correlation between salt intake and blood pressure (reviewed in ref. 2). This discrepancy could be attributed to variations in the consumption of other minerals such as potassium, magnesium, and calcium. It has been suggested that availability of adequate amounts of calcium stabilizes the cell membrane (such as endothelial and smooth muscle cell membranes), blocks its own entry into the cells, and makes arterial smooth muscle cells less likely to contract [2]. It was reported that hypertensives consume less sodium than those with normal blood pressure and significantly less calcium, potassium, vitamin A, and vitamin C [2]. In SHR (spontaneously hypertensive rat) showed a lower level of sodium contents in the RBC and a higher activity of the sodium pump. These studies led to the suggestion that the amounts of dietary calcium might regulate blood pressure by changing the sodium pump of the cell membrane in SHR. It is likely that it is not the calcium alone but calcium in conjunction with other ions such as sodium and potassium that relax the arterial smooth muscle cells. These results imply that it is likely that a balance between all the ions that is more important than calcium or for that matter any one ion in isolation that regulates the smooth muscle contraction and the development of hypertension [2].

It is well documented that increased consumption of high calorie diet or high fat diet (HFD) enhance the risk of development of insulin resistance, obesity, T2DM and HTN. Similarly, high dietary content of trans-fats and saturated fats enhance the incidence of insulin resistance, obesity and T2DM. In contrast to this, adequate exercise is beneficial in reducing the risk of insulin resistance, obesity, T2DM and HTN and consequently improve cardiovascular health. In addition, deficiency or sub-optimal intake of minerals, trace elements and vitamins increase the risk of development of obesity and T2DM [2]. Of all the dietary factors, perhaps, salt plays a dominant role in the pathobiology of insulin resistance, HTN and T2DM including cardiovascular health. In South Asian countries where salt intake is high, it may have a dominant role in the development of insulin resistance, HTN, T2DM and cardiovascular diseases. Despite this close association, the exact mechanism(s) by which salt plays a significant role in these diseases is not clear. It is suggested that high salt intake induces low-grade systemic inflammation, decreases NO and PGI2 generation, and thus, participates in the pathogenesis of these diseases. Based on the current evidence, it is proposed that high salt intake enhances the generation of TH17 cells that secrete enhanced amounts of IL-17, a pro-inflammatory molecule; suppresses the production of anti-inflammatory bioactive lipids (such as PGE1, PGI2, lipoxin A4, resolvins, protectins, and maresins). This imbalance between pro-inflammatory IL-17 (due to enhanced generation and activity of TH17 cells) and decreased production of anti-inflammatory bioactive lipids that have a negative regulatory control on IL-6 and TNF-α, results in an increase in the production of pro-inflammatory IL-6 and TNF-α, which induce the production of reactive oxygen species (ROS), reduce the generation of nitric oxide (NO) and enhance sympathetic activity. These events result in an increase in insulin resistance and subsequent development of HTN and T2DM. PGE1, PGI2, LXA4, resolvins, protectins and maresins, and NO are vasodilators and a decrease in their production/action results in an increase in peripheral vascular resistance and development of HTN [11]. Several studies showed that LXA4, resolvins, protectins and NO have anti-diabetic actions [12–17]. These results suggest that inflammation and immunological events participate in the development of HTN and DM implying that these two diseases have several common overlapping pathophysiological events.

Increased salt (sodium chloride) intake enhances the induction of human TH17 cells by activating the p38/MAPK pathway and serum/ glucocorticoid-regulated kinase 1 (SGK1) [18] resulting in upregulation of the proinflammatory cytokines GM-CSF, TNF-α, and IL-2 leading to the development of inflammatory events seen in HTN and T2DM. Thus, increased intake of salt induced SGK1 expression promotes IL-23R expression which enhances TH17 cell differentiation and accelerates the development of inflammatory events that can be suppressed by potassium supplementation [19]. These results imply that the balance between salt and potassium needs to be maintained to regulate TH17-induced inflammatory events, vascular tone, insulin resistance and prevent development of HTN and T2DM [2, 11, 20–22].

### Hypertension and T2DM have overlapping features

Hypertension is common in many countries. It is estimated that the prevalence of hypertension among adults was 29.0% and was similar among men (30.2%) and women (27.7%) in USA. Approximately ~ 50% of patients with T2DM have and/or develop HTN that enhances risk of vascular diseases (including coronary heart disease: CAD; peripheral vascular disease, stroke) due to DM. The risk for cardiovascular disease (CVD) is ~ four-fold higher in those with both DM and HTN compared to the normotensive non-diabetics. DM enhances the risk for coronary heart disease (CAD), stroke and deaths from cardiovascular cause, including heart failure, cardiac arrhythmia, sudden death, hypertensive disease, and aortic aneurysms [8]. The enhanced risk of complications due to DM are common in those who also have HTN. Subjects who have both HTN and T2DM are generally older, have higher BMI and hypertriglyceridemia (and other lipid abnormalities). These patients have higher expression levels of NADPH oxidase and MnSOD in their peripheral blood mononuclear cells [23–26].

NADPH oxidase (nicotinamide adenine dinucleotide phosphate oxidase) is a plasma membrane-bound enzyme complex that is used by neutrophils to inactivate microorganisms by producing superoxide free radical. The NADPH oxidase complex is dormant under normal circumstances but is activated during respiratory burst. Superoxide anion kills bacteria directly by attenuating their superoxide dismutase (SOD) genes and generating hydrogen peroxide and other reactive oxygen species (ROS). These free radicals inactivate several metabolic enzymes, initiate lipid peroxidation, damage iron-sulphur clusters, and allows the generation of indiscriminate oxidants. Vascular NADPH oxidases are important players in vascular remodeling and disease. Thrombin, platelet-derived growth factor (PDGF), tumor necrosis factor-α (TNF-α), interleukin-1, oxidized LDL and arachidonic acid can activate NDPH oxidase [27–35]. In those with uncontrolled hypertension, there is increased generation of free radicals including superoxide anion by their peripheral leukocytes and endothelial cells that can be attenuated by various antihypertensive drugs [11, 36–39]. It has been suggested that excess superoxide produced scavenges endothelial nitric oxide that, in turn, leads to the increased vascular smooth muscle contraction and hence to the elevated total peripheral resistance and development of HTN [40]. Similar increased production of free radicals has also been reported in those with T2DM and coronary heart disease [41–46].

Manganese-dependent superoxide dismutase (MnSOD) is an enzyme which is a member of the iron/manganese superoxide dismutase family that binds to the superoxide byproducts of oxidative phosphorylation and converts them to hydrogen peroxide and diatomic oxygen (O_2_). Thus, MnSOD clears reactive oxygen species (ROS) and protects cells from apoptosis. Thus, MnSOD protects various cells/tissues from oxidative stress induced by various agents and from inflammatory cytokines [47, 48]. These results suggest that a balance need to be maintained between ROS and MnSOD (SOD exists in three different isoforms: SOD1, SOD2, and SOD3. SOD1 is distributed throughout the cell cytoplasm, nucleus and in the lumen between outer and inner membranes of mitochondria, SOD2 isoforms are in matrix of mitochondria, while SOD3 is found mostly extracellularly) such that inappropriate apoptosis of cells would not occur due to excess production of ROS.

Since, ROS generation is increased in both T2DM and HTN [36–44], it may explain as to why the risk of development of T2DM is high in those who have HTN [49–53]. These results [49–53] suggest that HTN and T2DM may have some common pathophysiological basis including an increase in free radical generation. Both T2DM and HTN are considered as low-grade systemic inflammatory conditions since their plasma concentrations of tumor necrosis factor-α (TNF-α), interleukin-6 (IL-6), and C-reactive protein (CRP) are higher compared to normal healthy control [53–57]. High salt intake enhances the risk of development of HTN and T2DM [58–60]. In addition, increased generation of ROS and low levels of plasma NO is present in this situation [11, 36–40]. Furthermore, high salt intake induced HTN is associated with increased sympathetic activity (and so enhanced production of noradrenaline and adrenaline), augmentation of intrarenal angiotensin II production, enhanced oxidative stress and inflammatory cytokines [61–64]. The pro-inflammatory action of enhanced sympathetic activity [65] may counteract the anti-obesity action of sympathetic nervous system. In contrast to this, vagal acetylcholine, the principal neurotransmitter of the parasympathetic nervous system, has potent anti-inflammatory actions [66, 67]. In this context, the beneficial action of exercise is interesting. Sympathetic activity, ROS, plasma IL-6 and TNF-α levels are increased during exercise that are pro-inflammatory in nature [68–75]. But regular exercise enhances parasympathetic activity and augments vagal tone that results in increased generation of acetylcholine that has anti-inflammatory actions [66, 67, 69, 70, 76, 77]. Interestingly, regular exercise causes a gradual but sustained fall in the generation of IL-6 and TNF-α and enhances the production of MnSOD leading to an increase in endogenous antioxidant capacity [73–75]. This ultimately results in decrease of blood pressure, and protection from the development of T2DM and CHD [69, 70, 76, 77]. Thus, exercise is anti-inflammatory in nature [69, 70].

### Sodium and potassium modulate inflammation

High intake of sodium causes hypertension by volume expansion, altering the renin–angiotensin–aldosterone system (RAS), reducing endothelial NO generation, enhancing the formation of asymmetrical dimethyl arginine (ADMA), oxidative stress secondary to excessive production of ROS, inflammation, impaired insulin-mediated vasodilatation, increased sympathetic nervous system (SNS) activation, dysfunctional immune responses, and abnormal renal handling of sodium [2, 9, 11, 59–64]. Similar abnormalities are also present in T2DM [41–44, 57–61, 9, 23, 49]. These results are in support of the observation that expression of NADPH oxidase is increased in those with obesity, higher BMI and HTN and T2DM [23, 24]. This enhanced generation of ROS in those with T2DM and HTN can be attributed to an increase in the concentrations of pro-inflammatory cytokines IL-6, TNF-α and IL-17. In addition, angiotensin-II is a potent inducer of inflammation and generation of ROS that may explain as to how increase in intake of sodium-induced renin-angiotensin-aldosterone system induces inflammation and ROS induction [78–81]. Angiotensin-II enhances the formation of ADMA (asymmetrical dimethylarginine), a potent inhibitor of endothelial nitric oxide generation [81]. Thus, high salt intake reduces NO generation, increases ROS generation, decreases microvascular antioxidant enzymes (especially Cu/Zn SOD activity) and thus, contributes to enhanced peripheral vascular resistance and development of hypertension [82–86].

Recent studies revealed that increased salt (sodium chloride) intake augments the activity of TH17 cells via the p38/MAPK pathway and serum/glucocorticoid-regulated kinase 1 (SGK1) [18] that results in upregulation of GM-CSF, TNF-α and IL-2 levels, which are all pro-inflammatory cytokines that may account for the pro-inflammatory status seen in HTN and T2DM. This ability of salt to augment pro-inflammatory events can be suppressed by adequate potassium supplementation [19, 87, 88]. This indicates that a balance needs to be maintained between salt and potassium to suppress inappropriate induction of TH17-induced inflammatory events so that an increase in vascular tone and insulin resistance does not occur that ultimately leads to the development of HTN and T2DM. Thus, maintenance of balance among sodium, potassium, magnesium, and calcium in the physiological range prevents the development of HTN and T2DM [2].

**Fig. 1:**
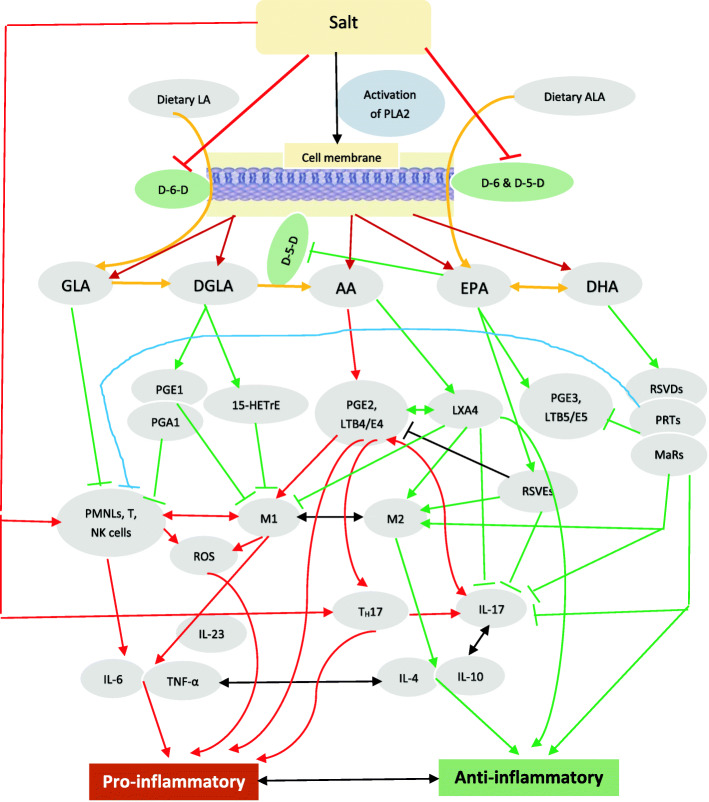
Scheme showing EFA metabolism, interaction, and feedback regulation among immunocytes, cytokines, various bioactive lipids (BALs) and inflammation. 15-HETrE = 15-(S)-hydroxy-8,11,13-eicosatrienoic acid. M1 and M2 macrophages. Red lines indicate pro-inflammatory events; Green lines indicate anti-inflammatory events. For further details see the text

Magnesium is a co-factor needed for the normal physiological activity of desaturases [2, 89, 90] that are needed for the conversion of dietary essential fatty acids (EFAs): linoleic acid (LA) and alpha-linolenic acid (ALA) to their respective long-chain metabolites that have important actions in the pathobiology of HTN and T2DM (see Fig. 1 for metabolism of EFAs and interaction among EFAs and their metabolites and other soluble mediators involved in HTN and T2DM). In addition, sodium and potassium influence the metabolism of EFAs by virtue of their ability to alter (sodium enhances whereas potassium decreases) the concentrations of angiotensin-II. Increased formation of angiotensin-II augments the generation of ROS that inactivates desaturases and thus, decreases the formation of their long-chain metabolites (GLA, DGLA, AA, EPA and DHA) [91, 92] some of which are the precursors of lipoxins, resolvins, protectins and maresins that have vasodilator, platelet antiaggregatory, anti-diabetic and anti-inflammatory actions [93–96]. Sodium inhibits whereas potassium enhances the activities of desaturases and thus, influence the conversion of dietary LA and ALA to their respective long-chain metabolites GLA, DGLA, AA and EPA and DHA, the precursors of pro- and anti-inflammatory prostaglandins (PGs), thromboxanes (TXs), leukotrienes (LTs), lipoxins resolvins, protectins and maresins. Sodium enhances the formation of PGs, TXs and LTs and blocks the formation of lipoxins, resolvins, protectins and maresins at least, in part, by inhibiting the generation of AA, EPA and DHA, whereas potassium shows opposite actions [97–106]. This may explain as to why excess salt consumption leads to pro-inflammatory status and development of HTN and T2DM.

### Potassium, sodium, and T cells

Potassium ions are not only the most abundant cation in the body but are also capable of regulating T cell function. Most of the potassium (~98%) is intracellular and only (~2%) a small portion of it is extracellular. In view of its actions on the cardiac muscle, the extracellular K^++^ concentrations need to be tightly controlled. Under normal physiological conditions, sodium and potassium ions have opposite actions. Increased sodium chloride concentrations enhance the induction of TH17 cells by activating the p38/MAPK pathway and serum/glucocorticoid-regulated kinase 1 (SGK1) [18, 19, 87, 88, 107–112]. These TH17 cells secrete large amounts of pro-inflammatory cytokines GM-CSF, TNF-α and IL-2 that coincided with severe forms of autoimmune disease such as experimental autoimmune encephalomyelitis (EAE). It is noteworthy that other dietary factors such as high-fat and cholesterol, high-protein, high-sugar, and frequent consumption of processed and 'fast foods' that contain significantly high amounts of salt (sodium chloride) not only promote obesity, metabolic syndrome, and cardiovascular diseases but also augment T_H_17 cell generation and production of IL-17 cytokine and thus, promote autoimmune diseases. Thus, increased dietary salt, high fat diet and high sugar all enhance the risk of developing obesity, HTN and T2DM and autoimmune diseases (such as rheumatoid arthritis, lupus, multiple sclerosis) through the induction of pathogenic TH17 cells [18, 107–113] whereas elevated intracellular K^+^ concentration suppresses T cell function by inhibiting Akt and mTOR protein kinases [19, 88–90, 114, 115]. Hence, maintaining the balance between Na^+^ and K^+^ across the cell membrane is critical not only for T cell function but also for other cells such as endothelial cells to prevent inappropriate production of pro-inflammatory cytokines (endothelial cells also produce cytokines [116]). Thus, dietary salt and K^+^ intake modulate GM-CSF, TNF-α, IL-2 and IL-17 production and thus, play a role in the pathobiology of HTN and T2DM.

### HTN as an immunological disorder

The observation that salt enhances the production of IL-17, a pro-inflammatory molecule, implies that HTN could be an immunological disorder. Increased salt intake induced elevation in plasma angiotensin-II levels [62] that, in turn is known to enhance IL-6 secretion and other pro-inflammatory molecules such as PGE2, NF-kB and IL-17 [24, 63–65, 78–81]. It was reported that the basal plasma levels of IL-6 were higher in those with HTN compared to control. These results suggest that HTN is associated with pro-inflammatory status and angiotensin-II is a pro-inflammatory molecule.

**Fig. 2:**
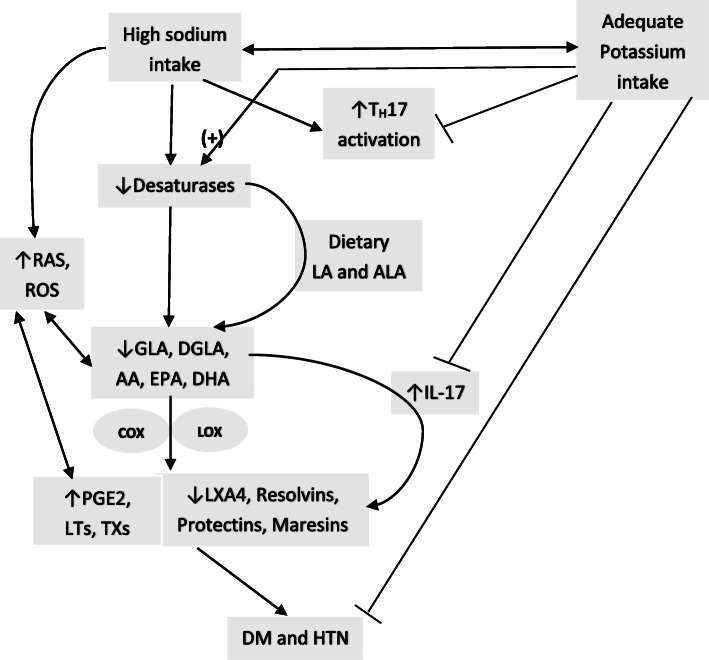
Scheme showing the effect of high sodium intake and potassium on the activity of desaturases, and formation of their metabolites. High sodium intake inhibits the activity of desaturases and the conversion of dietary LA and ALA to their respective metabolites. This results in decreased formation of vasodilator, and anti-inflammatory LXA4, resolvins, protectins and maresins due to the deficiency of their respective precursors and an increase in PGE2, LTs and TXs are pro-inflammatory in nature. Sodium enhances TH17 activation and enhances IL-17 formation and release whereas potassium inhibits these actions. IL-17 has pro-inflammatory actions and enhances ROS generation. For further details see text

**Fig. 3:**
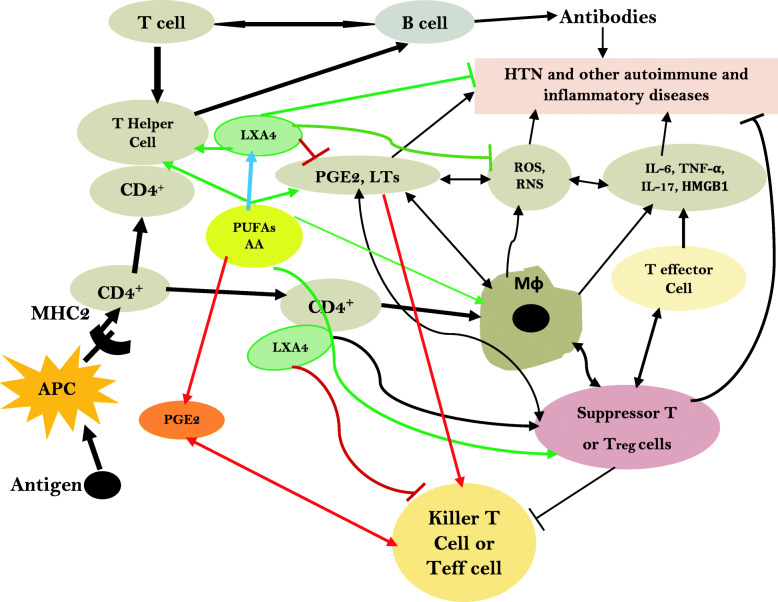
Scheme showing interaction(s) among T and B cells and macrophages and their relationship to various diseases. Possible role of PUFAs and their metabolites in these events is also depicted. Antigen-presenting cells (APCs) present antigen on their Class II MHC molecules (MHC2). Helper T cells recognize these, with the help of their expression of CD4 co-receptor (CD4+). The activated helper T cell release cytokines and other stimulatory signals that stimulate the activity of macrophages, killer T cells and B cells. The activated T cells, B cells and macrophages produce eicosanoids, ROS, NO and cytokines that eliminate the invading microorganisms, intracellular pathogens and/or cause autoimmune diseases depending on the regulation or inappropriate function of T suppressor cells. Decreased production of LXA4/resolvins/protectins/maresins and abnormal EFA metabolism leads to alterations in the production of ROS, IL-17, IL-6, TNF-α and antibodies resulting in development of HTN and DM. This diagram is an abridged form of the actual interactions that are much more complex

**Fig. 4:**
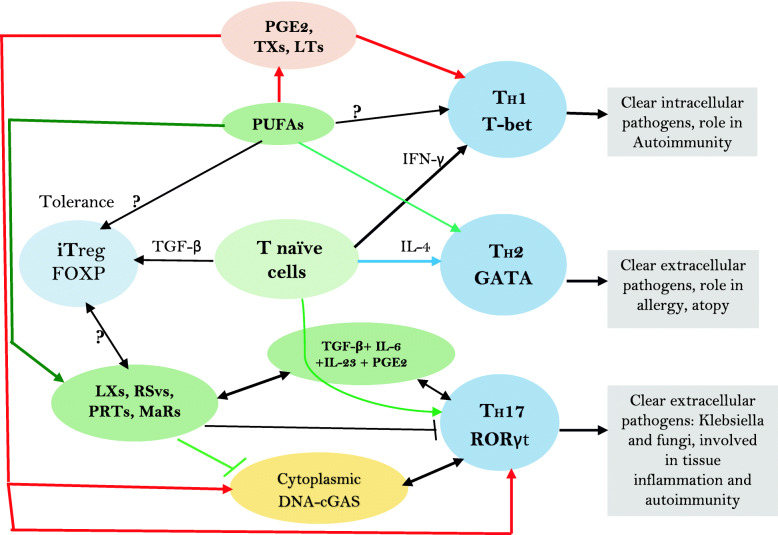
Factors controlling formation of different subsets of T helper cells. LXs = Lipoxins; RSvs = Resolvins; PRTs = Protectins; MaRs = Maresins. Naive CD41 T cells differentiate into 3 types of subsets of T helper cells: TH1, TH2 and TH17. TGF-β converts naive T cells into FOXP3-expressing induced Treg (iTreg) cells. T helper cell differentiation requires specific transcription factors as master regulators, which include T-bet, GATA3 and ROR-γt. Terminally differentiated T helper cells produce specific effector cytokines to bring about their distinct effector functions. TGF-β, retinoic acid or cytokines: IL-6, IL-1, IL-23, or IL-27 produced by the innate immune system’s immature or activated dendritic cells (DCs) dictate how a naive T cell develops into a FOXP31 Treg cell, a TH17 cell or otherwise. PGE2 through its receptor EP4 on T cells and dendritic cells facilitates TH1 cell differentiation and amplifies IL-23–mediated T_H_17 cell expansion, whereas EP4-selective antagonist inhibits TH1 and TH17 cells and suppresses autoimmune diseases. GLA, AA, EPA, DHA, lipoxins, resolvins, protectins, maresins and prostaglandins, leukotrienes and thromboxanes influence macrophage and other immunocytes’ phagocytosis, motility and ability to alter ROS generation and thus, regulate inflammation and immune response

Furthermore, angiotensin-II mediates induction of HTN by acting on T effector (Teff) cells. Under normal physiological conditions, T regulatory cells (Tregs) suppress Teff lymphocytes. In experimental animals, infusion of angiotensin-II (1 μg/kg/minute subcutaneous) for 14 days increased systolic blood pressure by more than 40 mm Hg, enhanced NADPH oxidase activity in aorta and heart by more than 1.5 fold, impaired acetylcholine vasodilatory action by ~70% compared to control and enhanced vasculature stiffness and mesenteric artery vascular cell adhesion molecule expression by more than 2 fold and aortic macrophage and T cell infiltration, events that could be reversed by Treg cell adaptive transfer. Angiotensin-II induced decrease in Foxp3^+^ in renal cortex that was reverted to near normal by Treg cells transfer. These results suggest that angiotensin-induced hypertension and other features are due to its pro-inflammatory action mediated by Teff cells and this can be reversed or prevented by Treg cells that have anti-inflammatory actions [117–125]. Thus, it is opined that angiotensin-II induced blood pressure elevation is due to its ability to induce oxidative stress, endothelial dysfunction, and activation of Teff cell function. It is known that Treg cells deficiency is associated with several autoimmune diseases [126]. This is further supported by the observation that IL-17 levels are increased in subjects with type 1 DM, lupus, and multiple sclerosis [127–130]. These results coupled with the reports that circulating levels of IgG and IgM antibodies are elevated in patients with essential and pregnancy-related hypertension that target G-protein coupled receptors and ion channels including AT1 receptors, α1-adrenoceptors, β1-adrenoceptors, and L-type voltage operated Ca(2+) channels imply that these antibodies play a critical role in the events that are important for regulating blood pressure including modulation of vascular tone, cardiac output, and/or Na(+)/water reabsorption in the kidneys [131]. Since normally there is a close interaction between T and B cells in the regulation of immune response, it is reasonable to suggest that both T and B cells have a regulatory role in the pathobiology of HTN and T2DM and other inflammatory diseases including type 1 DM. In this context, it is noteworthy that EFAs and their metabolites have a regulatory role in the generation and action of T and B cells [132–144] (see Figs. 2, 3 and 4). At this juncture, it is reasonable to argue that not all hypertensives have increased salt intake except those who have salt sensitive HTN. It is likely that in those who do not have salt sensitive HTN may have other abnormalities such as relatively decreased intake of potassium, magnesium and calcium and decreased formation of downstream metabolites of EFAs such as GLA, AA, EPA and DHA and lipoxins, resolvins protectins and maresins. These abnormalities may lead to increased formation of IL-17 secondary to the absence of negative feedback control exerted by potassium, magnesium, EFAs and their metabolites. This ultimately leads to enhanced generation of IL-17 by endothelial cells and various immunocytes leading to the onset of HTN [116, 145–149].

### EFAs in the pathobiology of HTN and T2DM

Dietary EFAs: cis-linoleic acid (LA, 18:2 n-6) and alpha-linolenic acid (ALA, 18:3 n-3) are essential for the survival of humans. EFAs are needed for integrity of skin, normal immune function, and inflammation. EFAs deficiency can result in death due to dehydration, desquamation of skin, poor wound healing, growth retardation, immunosuppression and increased infections, hepatic and splenic dysfunction and abnormal inflammation that eventually can lead to death. But EFAs deficiency is rare since they are present in almost all food that we consume. EFAs deficiency is common in those who are on total parenteral nutrition (when they do not receive enough EFAs in the parenteral fluids), suffer from long-standing and severe bowel disease, following massive bowel resection surgery, pancreatic surgery, and gastric bypass surgery and have significant cystic fibrosis [150–153]. Due to the awareness of importance of EFAs in health, now a days all patients receive EFAs supplementation and so is rare. The significance of EFAs resides in the fact that they are essential for normal skin function and to prevent water loss through skin since they are important for skin integrity and for normal immune response. EFAs form an important constituent of cell membrane and regulate cell membrane fluidity [153]. In addition, EFAs are converted into their long-chain metabolites namely gamma-linolenic acid (GLA, 18:3 n-6), dihomo-GLA (DGLA, 20:3 n-6) and arachidonic acid (AA, 20:4 n-6) from LA and eicosapentaenoic acid (EPA, 20:5 n-3) and docosahexaenoic acid (DHA, 22:6 n-3) from ALA by the action of enzymes delta-6- and delta-5-desaturases and elongases (see Fig. 3). DGLA is the precursor of 1 series prostaglandins (PGs), whereas AA is the precursor of 2 series PGs, thromboxanes (TXs) and 4 series leukotrienes (LTs). EPA is the precursor of 3 series PGs and TXS and 5 series LTs. PGs, TXs and LTs generally have pro-inflammatory actions but those formed from EPA are less pro-inflammatory compared to those formed from AA. In addition, PGE1 from DGLA, PGI2 and PGJ2 from AA have anti-inflammatory actions. AA is the precursor of lipoxin A4 (LXA4), a potent anti-inflammatory compound. EPA is the precursor of resolvins of E series, whereas resolvins of D series, protectins and maresins are derived form DHA. Resolvins, protectins and maresins are all potent anti-inflammatory compounds. It is evident from this discussion that EFAs are the precursors of a variety of pro and anti-inflammatory compounds that play a significant role in inflammation, wound healing, immune response and protecting the body from various bacterial, viral, fungal, and parasitic infections [153]. It is note worthy that PGs, TXs, LTs, LXA4, resolvins, protectins and maresins modulate vascular tone, regulate insulins secretion, and have significant role in the modulation of actions of leukocytes, macrophages, monocytes, T cells and B cells [154–157]. In view of these actions, EFAs and their metabolites have a critical role in the regulation of blood pressure and pathobiology of both type 1 and type 2 diabetes mellitus [15, 93, 158–163]. One of the actions of EFAs and their metabolites include their ability to modulate secretion and action of cytokines, ROS generation and T cell function (see Fig. 2).

### EFAs and their metabolites regulate T cells development and its relevance to HTN and T2DM

On exposure to different stimuli, naïve T cells get activated, form three distinct effector TH subsets: TH1 cells produce IFN-γ; TH2 cells produce IL-4, IL-13 and IL-25; the third subset of TH cells known as TH17 cells produce IL-17. TH17 cells also produce IL-21 and IL-22. TGF-β, IL-6, IL-21, IL-23, and IL-1β have a role in the formation of TH17 cells. High concentrations of TGF-β inhibit IL-6-induced IL-22 expression, whereas a combination of TGF-β and IL-6 induce generation of IL-17 by TH17 cells. IL-22 production by TH17 cells need the co-operation of IL-23. Thus, IL-22 is the end point effector cytokine secreted by TH17 cells [164–171] (see Figs. 1, 2 and 3).

IL-17, a pro-inflammatory cytokine, is secreted by T helper 17 cell in response to stimulation by IL-23. IL-17 stimulates the induction of chemokines and thus, monocytes and neutrophils are recruited to the site of inflammation. IL-17 acts in conjunction with TNF and IL-1 and plays a critical role in autoimmune diseases and allergic responses. IL-17 enhances the production of IL-6, G-CSF, GM-CSF, IL-1β, TGF-β, TNF-α, chemokines (including IL-8, GRO-α, and MCP-1), and PGE2 by macrophages and other cells [172]. TH17 is needed for protection against Gram-positive and Gram-negative organisms and fungi [172] (see Fig. 4).

TH17 development is regulated by the transcription factor, the orphan nuclear receptor ROR-ct and TGF-β plus IL-6. Furthermore, Treg cells and TH17 cells have a reciprocal relationship between them. This is supported by the observation that IL-2, a growth factor for Treg cells, inhibits the generation of TH17 cells, whereas lack of IL-2 reduces the number of Treg cells and increases TH17 cells and leads to the development of inflammatory diseases that can be suppressed Treg cells. IL-4, IL-25, IL-27 and IFN-γ inhibit the expansion of TH17 cells [166–172].

But surprisingly antibodies against IL-17 are not effective against inflammatory bowel disease, an autoimmune disease. In contrast, targeting IL-23 is effective [171]. PUFAs (especially EPA/DHA), lipoxins, resolvins, protectins and maresins inhibit IL-23 and IL-17 and hence are expected to prevent salt and HFD and carbohydrate-induced HTN and T2DM (see Fig. 4).

Several studies revealed that EFAs and their metabolites can regulate blood pressure and prevent development of HTN, at least, in part by restoring IL-17 and Treg balance, decreasing the formation of angiotensin-II and suppressing the expression of angiotensin-II receptors [141, 173–181]. We showed that GLA, DGLA, AA, EPA and DHA can prevent the development of both type 1 and type 2 DM by alloxan and streptozotocin in experimental animals [158, 160, 163, 182]. Of all the fatty acids tested, AA was found to be the most potent in preventing type 1 and type 2 DM due to its conversion to LXA4 [93]. It was found that resolvins and protectins also have anti-diabetic actions [183–185].

PGE2, derived from AA, induces the expression of IL-23R in naïve CD4^+^ T cells. TXA4, a pro-inflammatory molecule derived from AA, also facilitates IL-17A production from Vγ4^+^ γδ T cells [186–193]. Lipoxins, resolvins, protectins and maresins suppress IL-17 and IL-23 production, whereas PGE2 and TXA4 have opposite actions [186–193]. But, in an occasional instance, PGE2 suppresses the production of IL-23 and IL-12 and thus bring about its anti-inflammatory action [194]. But, in general, PGE2 and LTs, enhance the production of IL-17 and IL-23, whereas lipoxins, resolvins, protectins and maresins suppress their production [186–193] (see Figs. 1, 2, 3 and 4).

## Conclusions and therapeutic implications

It is evident from the preceding discussion that increased salt intake (and perhaps, in a similar fashion-carbohydrate/high fat diet or energy dense food) results in an: (i) increase in IL-17 production; (ii) decrease in the activity of desaturases leading to low levels of GLA, DGLA, AA, EPA and DHA and their anti-inflammatory, vasodilator, and platelet anti-aggregator metabolites LXA4, resolvins, protectins and maresins that have the ability to suppress IL-16, IL-6, IL-17 and TNF-α production; (iii) increase in PGE2, LTs, TXA2 production that have pro-inflammatory action [195, 196]; (iv) increase in the production of angiotensin-II that is pro-inflammatory in nature-events that facilitate the occurrence of HTN and T2DM. We observed that PUFAs can suppress the activity of ACE (angiotensin converting enzyme) [197] and thus, inhibit the formation of angiotensin-II, a pro-inflammatory and IL-17 formation stimulatory molecule. In addition, nitric oxide is a potent suppressor of ACE activity [197] and antagonizes the pro-inflammatory and vasoconstrictor action of angiotensin-II. PUFAs and their metabolites especially, PGE1, PGI2, LXA4, resolvins, protectins and maresins augment the production of NO [198–201] and thus, are of benefit in the prevention of HTN and DM. Despite the evidence presented above, it is not without controversy. For instance, it was reported that high-salt intake induces severe hypertension in WBN/Kob diabetic fatty (WBKDF) rats, whereas plasma glucose levels were significantly increased in WBKDF-NS (normal salt) but not in WBKDF-HS (high salt) rats. Even HOMA-IR (insulin resistance) in WBKDF-HS was significantly lower compared with that in WBKDF-NS that coincided with an increase in plasma adiponectin level in WBKDF-HS group. This paradoxical response shown by WBKDF-HS group to high salt supplementation in the form of reduced hyperglycemia and insulin resistance in WBKDF rats has been attributed to increased plasma levels of adiponectin [202]. In contrast to this, WBKDF-HFD (high fat diet) and WBKDF-FRD (fructose rich diet) rats exhibited aggravated obesity and dyslipidemia compared with WBKDF rats fed standard diet (STD). Paradoxically, hyperglycemia developed in WBKDF-STD rats was significantly inhibited in WBKDF-FRD rats, but not in WBKDF-HFD rats [203]. These results suggest that the response of WBKDF rats to high salt intake and high fat diet depends on other homeostatic mechanisms such as adiponectin and secretion of NO, and response of the autonomic nervous system (including secretion of adrenaline and noradrenaline and acetylcholine). This argument is supported by the observation that WBKDF rats when fed with high salt not only had significant elevation of systolic blood pressure (SBP) but also showed enhanced phenylephrine-induced contractions of isolated thoracic aortic rings significantly reduced relaxation to acetylcholine- and nitroprusside and higher plasma concentrations of 8-iso-prostaglandin F_2α_ (a metabolite of PGI2, a vasodilator, platelet anti-aggregator and anti-inflammatory molecule) [204]. The reduced responses to phenylephrine, acetylcholine and nitroprusside could be attributed to higher constitutional production of NO and PGI2 in WBKDF rats that is exaggerated by high salt and high fat diet. This is supported by the observation that NO can prevent hyperglycemia [205, 206]. It is noteworthy that adiponectin enhances eNO generation [207, 208] and augments insulin action [209, 210] explaining why diabetes mellitus is ameliorated in WBKDF-HS group.

Sodium-glucose cotransporter 2 inhibitors (SGLT2i) are hypoglycemic drugs that target SGLT2, the major glucose transporter in the kidney responsible for about 90 percent of glucose reabsorption from primary urine [8]. SGLT2i reduce glycosylated hemoglobin, body weight, blood pressure, plasma volume, and improve cardiac energy metabolism. The beneficial actions of SGLT2i are due to their ability to inhibit vascular inflammation, reduce oxidative stress, reverse endothelial dysfunction, reduce foam cell formation and prevent platelet activation [211], which are all pro-inflammatory events. It is interesting to note that SGLT2i empagliflozin acts via AMPK/mTOR pathway to enhance autophagy of hepatic macrophages and thus, prevents NAFLD-related liver injury. Furthermore, empagliflozin inhibits IL-17/IL-23 axis and thus, mediates its anti-inflammatory actions [212, 213]. This lends support to the concept that both salt and glucose bring about their pro-inflammatory actions by enhancing the production of IL-17.

In this context, it is noteworthy that regulatory T cells (Tregs) express FOXP3, which is needed for natural Tregs (nTregs) along with TGFβ and retinoic acid for induced Tregs (iTreg). Interestingly, the signature transcription factor for Th17 cells, RORγt, is induced by TGFβ, thus linking the differentiation of the Treg and TH17 lineages. FOXP3 can inhibit RORγt function and drive Treg differentiation. However, when the cell receives a signal from IL-6, FOXP3 function is inhibited and the differentiation pathway is induced. Thus, the balance between FOXP3 and RORγt function determines CD4 T cell fate and the type of immune response that will be generated [214]. It is important to note that PGE2, LTs, and other bioactive lipids regulate the expression of FOXP3 and thus, regulate/control the generation of TH17 cells, their ability to secrete IL-17; Tregs and thus, modulate inflammatory process [179, 181, 215–232] (see Figures 3 and 4).

Based on these results, it is suggested that in addition to reducing the intake of salt, carbohydrate and energy dense foods, it is worthwhile to supplement various EFAs, especially AA/EPA/DHA, their co-factors that are essential for the normal physiological action of desaturases such as vitamin C, folic acid, B1, B6, B12, zinc, and magnesium to prevent the development of HTN and T2DM and to inhibit their progression if they are already present.

## Data Availability

Not applicable

## Abbreviations

BALs: Bioactive lipids (these are LA, ALA, GLA, DGLA, AA, EPA, DHA, lipoxins, resolvins, protectins, maresins, prostaglandins, leukotrienes, thromboxanes as defined in the present discussion)
EFAs: Essential fatty acids
ALA: Alpha-linolenic acid
AA: Arachidonic acid
CVD: Cardiovascular disease
cGAS-STING: cyclic GMP-AMP synthase linked to stimulator of interferon genes
COX: Cyclo-oxygenase
DGLA: Dihomo-GLA
DHA: Docosahexaenoic acid
EPA: Eicosapentaenoic acid
GLA: Gamma-linolenic acid
GM-CSF: Granulocyte-macrophage colony stimulating factor
HFD: High fat diet
HTN: Hypertension
IL: Interleukin
IFN: Interferon
LA: Linoleic acid
LXA4: Lipoxin A4
LTs: Leukotrienes
LOX: Lipoxygenase
MnSOD: Manganese superoxide dismutase
NADPH: Nicotinamide adenine dinucleotide phosphate
NO: Nitric oxide
PLA2: Phospholipase A2
PNS: Parasympathetic nervous system
PGs: Prostaglandins
ROS: Reactive oxygen species
SNS: Sympathetic nervous system
STZ: Streptozotocin
TXs: Thromboxanes
T2DM: Type 2 diabetes mellitus
TNF-α: Tumor necrosis factor-α
T_H_ cells: T helper cells
Teff cells: T effector cells
